# Targeting Mitochondria for Treatment of Chemoresistant Ovarian Cancer

**DOI:** 10.3390/ijms20010229

**Published:** 2019-01-08

**Authors:** Edith Emmings, Sally Mullany, Zenas Chang, Charles N. Landen, Stig Linder, Martina Bazzaro

**Affiliations:** 1 Masonic Cancer Center and Department of Obstetrics, Gynecology and Women’s Heath, University of Minnesota, Minneapolis, MN 55455, USA; edieb.543@gmail.com (E.E.); smullany@umn.edu (S.M.); zechang@umn.edu (Z.C.); 2 Department of Obstetrics and Gynecology, University of Virginia, Charlottesville, VA 22908, USA; CL3NJ@hscmail.mcc.virginia.edu; 3 Cancer Center Karolinska, Department of Oncology and Pathology, Karolinska Institute, S-171 76 Stockholm, Sweden; 4 Department of Medical Health Sciences (IMH), Linköping University, S-751 85 Linköping, Sweden

**Keywords:** ascites, OXPHOS, mitochondrial inhibitor, chemoresistant ovarian cancer, SWI/SNF complex

## Abstract

Ovarian cancer is the leading cause of death from gynecologic malignancy in the Western world. This is due, in part, to the fact that despite standard treatment of surgery and platinum/paclitaxel most patients recur with ultimately chemoresistant disease. Ovarian cancer is a unique form of solid tumor that develops, metastasizes and recurs in the same space, the abdominal cavity, which becomes a unique microenvironment characterized by ascites, hypoxia and low glucose levels. It is under these conditions that cancer cells adapt and switch to mitochondrial respiration, which becomes crucial to their survival, and therefore an ideal metabolic target for chemoresistant ovarian cancer. Importantly, independent of microenvironmental factors, mitochondria spatial redistribution has been associated to both tumor metastasis and chemoresistance in ovarian cancer while specific sets of genetic mutations have been shown to cause aberrant dependence on mitochondrial pathways in the most aggressive ovarian cancer subtypes. In this review we summarize on targeting mitochondria for treatment of chemoresistant ovarian cancer and current state of understanding of the role of mitochondria respiration in ovarian cancer. We feel this is an important and timely topic given that ovarian cancer remains the deadliest of the gynecological diseases, and that the mitochondrial pathway has recently emerged as critical in sustaining solid tumor progression.

## 1. Introduction

The most recent global statistic estimates 295,414 newly diagnosed cases of ovarian cancer every year and 184,799 annual death from this disease [1]. Such a high mortality rate is primarily attributed to the fact that early disease produces absent or nonspecific symptoms, leading to delayed diagnoses until late stages. Greater than 75% of ovarian cancer cases go undetected until an advanced stage, which is difficult to treat effectively. The 5-year survival rate of late stage ovarian cancer remains around 30%, in spite of novel chemotherapy regimens such as intraperitoneal delivery and targeted therapies including poly (ADP-ribose) polymerase (PARP) inhibitors, anti-angiogenics [2]. Standard treatment consists of tumor debulking surgery followed by platinum and paclitaxel chemotherapy. Patients typically tolerate the treatment well and go into remission, but disease recurrence is common. Chemoresistance in ovarian cancer is due to both the intrinsic ability of ovarian cancer cells to resist chemotherapy and the ovarian cancer tumor microenvironment. The latter, which is composed by extracellular matrix (ECM), cancer-associated fibroblasts (CAF) and immune and endothelial cells, provides a positive feedback for ovarian cancer to thrive by increasing cancer cells’ proliferation, promoting angiogenesis, and help remodeling the extracellular matrix so to create space for the tumor to grow [3,4].

Due to these complexities, effective treatment of recurrent and chemotherapy-resistant disease remains a significant challenge in the management of epithelial ovarian cancer.

Recurrence arises when cell populations evade the effects of chemotherapy. Non-proliferative, or quiescent, cancer cells are resistant to paclitaxel and carboplatin. Although a general problem for all ovarian cancers, recurrence is variable among subtypes. The majority (90%) of ovarian cancers develop in the epithelium. Epithelial ovarian cancer (EOC) is what is referred to in generalizations of ovarian cancer. EOC is also made up of several subtypes, such as serous, endometrioid, clear cell, mucinous, and mixed [5]. Ovarian clear cell carcinoma (OCCC) has the highest rate of recurrence among EOC, suggesting a poor response to treatment [6]. Besides the epithelium, ovarian cancer can also originate from germ cells or stromal tissue, however these are diagnosed at earlier stages on average, and therefore have much higher survival rates. Small cell carcinoma of the ovary, hypercalcemic type (SCCOHT) is a rare ovarian cancer, typically found in young women, that is essentially untreatable [7]. This review primarily focuses on EOC. 

A consideration of the unique cellular and environmental features of chemoresistant ovarian cancer cells could shed light on potential therapeutic improvements targets. Depleted oxygen and glucose in the microenvironment of ovarian cancer limits the potential for cellular metabolic plasticity, which has only recently been appreciated as playing a key role in cancer progression and chemoresistance. This review focuses on the metabolic profiles and adaptations of chemoresistant ovarian cancer cells in order to exploit their unique characteristics and improve treatment outcomes. Historically, cancer cell metabolism has been understood through the Warburg effect, which describes a compensation for increased energy demand via upregulated glycolysis [8,9]. However, while Warburg effect is a necessary metabolic shift for cell division and proliferation [10], recent evidences suggests that mitochondria and Oxidative Phosphorylation (OXPHOS) are essential to bioenergetics, biosynthesis, and signaling in proliferative and quiescent cancer cells [11,12,13,14]. In fact, recent findings in ovarian cancer suggest that chemoresistant cell types have an elevated dependence on OXPHOS and sensitivity to OXPHOS inhibitors. We propose that tumor-selective inhibition of the electron transport chain (ETC) could eliminate these cells while allowing a therapeutic window for normal cells, therefore preventing ovarian cancer reoccurrence.

## 2. Microenvironment of Ovarian Cancer

**Figure 1:**
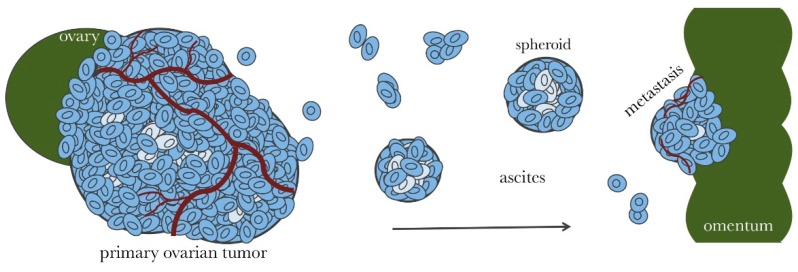
Ovarian cancer metastasis throughout the abdominal cavity. Proliferative ovarian cancer cells are indicated in dark blue. Adherent ovarian tumors (primary and metastatic) are irregularly vascularized, which results in cell populations that are deprived of necessary nutrients (indicated in light blue). These cells become quiescent making them resistant to chemotherapy. Ovarian tumors shed cells into the ascites fluid in the form of single cells or small cell clusters. Suspended ovarian cancer cells can aggregate and compact to form dense spheroids. Spheroids are proliferative on the surface, but contain a large population of quiescent cells within. Spheroids can invade surface tissues, such as the omentum, to access more nutrients and form metastatic tumors.

The majority of ovarian cancer patients are diagnosed after the disease has metastasized throughout the abdominal cavity [15]. At this stage, the average ovarian tumor is about the size of a lime [16]. A tumor of this size becomes irregularly vascularized resulting in poorly perfused, hypoxic regions [17]. While tumor hypoxia is widely appreciated, an occasionally overlooked detail is that avascular regions of tumors are not only hypoxic but also nutritionally compromised, resulting in a limited potential for cellular metabolic plasticity. Late stage and recurring ovarian cancer consist of solid tumors throughout the abdominal cavity. As tumors grow in size, they shed cells into the ascitic fluid [18]. Suspended ovarian cancer cells aggregate to form dense spherical clusters (spheroids), which invade surface tissues to form new tumors [19]. An accumulation of peritoneal ascitic fluid is more common in ovarian cancer than any other tumor type and thought to be the major conveyance for metastatic spread of ovarian cancer [18,20]. It causes bloating, decreased appetite, and discomfort and is one of the identifying symptoms of the disease. As depicted in Figure 1, the disease is usually contained to pelvic and peritoneal tissues for the majority of its progression. As a result, ovarian cancer subsists and spreads within a unique biochemical microenvironment compared to cancers that utilize the vasculature for metastasis [18,20]. Malignant ascites is hypoxic (~50% less soluble oxygen than the blood [21]) and has been reported to contain depleted levels of glucose [22].

A subset of cancer cells found throughout the ascites and spheroids in ovarian cancer are considered to have stem cell-like capabilities such as the ability to self-renew, differentiate, and initiate new tumors at low seeding [23,24]. Unlike somatic stem cells, ovarian cancer stem cells (CSCs) have been described to have rate of proliferation similar to other tumor cells, to be characterized by selective chemoresistance pathways and markers [25,26,27] and treating of the disease with chemotherapy, to enrich their prevalence [28]. Thus, CSCs pose a major hurdle for effective ovarian cancer treatment and are attractive targets for novel cancer therapies [29]. Depleted nutrients in the cellular microenvironment forces ovarian cancer cells to adapt their metabolic needs in order to survive creating vulnerability we hope to exploit.

## 3. Ovarian Cancer Spheroids and Their Characterization

Ovarian cancer spheroids suspended in the ascites can grow up to 500 µm in diameter [30]. Like tumors, spheroids of this size can develop chemical and nutritional gradients [17]. The outer cellular layer of a spheroid is proliferative and depends on the limited levels of glucose and oxygen in the ascites, but inside the spheroid, cells are dense and hypoxic [17]. Spheroids are considered a major vehicle for peritoneal metastasis [19] and are difficult to eliminate with chemotherapy. The main site for spheroid invasion is the omentum [18,31], which is a layer of fatty tissue that drapes over the abdomen and pelvis [20]. The omentum is made up of white adipocytes [32], which are actively involved in cytokine signaling [18]. Cytokines such as interleukin (IL)-6 and IL-8 attract spheroids to the omentum [31]. IL-6 may also contribute to the symptoms of nausea, vomiting, and anorexia, which compromise the availability of glucose [33]. Importantly, in vitro multicellular tumor spheroids (MCTS) have been used in drug screens to identify compounds that are effective against 3-dimensional spheroids including spheroids derived from ascites of ovarian cancer patients [34,35,36].

## 4. Microenvironment-Dependent Metabolism in Ovarian Cancer

The standard chemotherapy regimen for ovarian cancer patients is a combination of paclitaxel and carboplatin, which selectively eliminate rapidly proliferating cells [2]. In poorly vascularized areas of tumors, environmental factors will result in cells becoming quiescent [37], making them unresponsive to carboplatin and paclitaxel. In spheroids, for example, it is estimated that less than 40% of the cells are proliferative [38]. Hyperthermic intraperitoneal chemotherapy (HIPEC) has been used with some success to eliminate poorly vascularized tumors. A multicenter, open-label, phase 3 trial, conducted on ovarian cancer patients with stage III disease, showed that the use of HIPEC resulted in longer recurrence-free survival and overall survival than surgery alone suggesting that HIPEC could be used in the management of ovarian cancer as a part of first line therapy and second line therapy for recurrent disease [39].

Quiescent cancer cells in avascular regions, however, use mitochondrial OXPHOS for the majority of ATP production [10]. OXPHOS is able to function optimally at oxygen levels as low as 0.5% [11]. In ovarian cancer cells, increased dependence on OXPHOS corresponds to lower rates of proliferation and chemoresistance whereas increased glycolysis was associated with chemosensitivity [40]. Ovarian tumors show increased mtDNA copy numbers [41] and intact mitochondria isolated from human ovarian tumors have been shown to have substantive oxidative phosphorylating activities [42]. These observations are in agreement with recent studies of other types of cancer [43,44,45].

**Figure 2:**
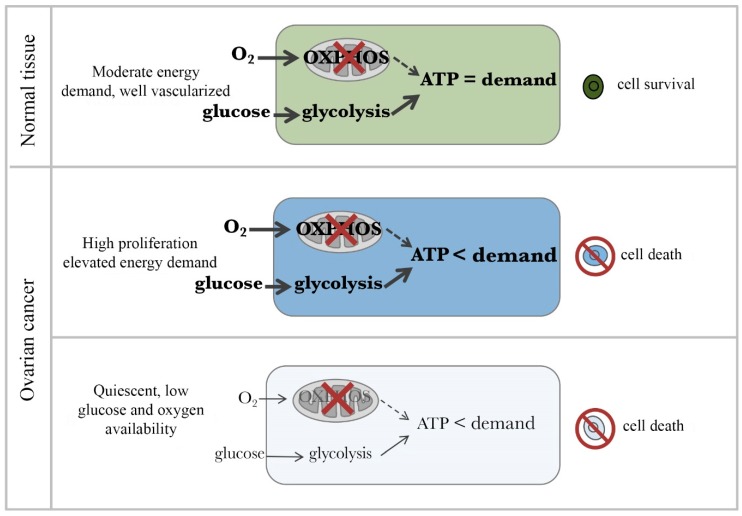
Therapeutic window for cancer cells in the presence of an OXPHOS inhibitor. (**Top**) Normal body cells have moderate ATP demand and adequate levels of oxygen and glucose. Thus, they survive in presence of OXPHOS inhibitors by upregulating glycolysis to meet their ATP demands. *Middle panel.* Highly proliferating cancer cells have extraordinarily high ATP demand and adequate levels of oxygen and glucose. Despite glycolytic pathway upregulation, OXPHOS inhibition causes cancer cells not to be able to meet their ATP demand and die. (**Bottom**) Quiescent cancer cells have low ATP demand but live in a highly compromised microenvironments characterized by low glucose and hypoxia. For these cells, inhibition of OXPHOS is lethal as there is not sufficient glucose to compensate for the loss of ATP production. Thicker arrows indicate relatively greater activity. Bold text indicates a relatively higher abundance of the item.

**Figure 3:**
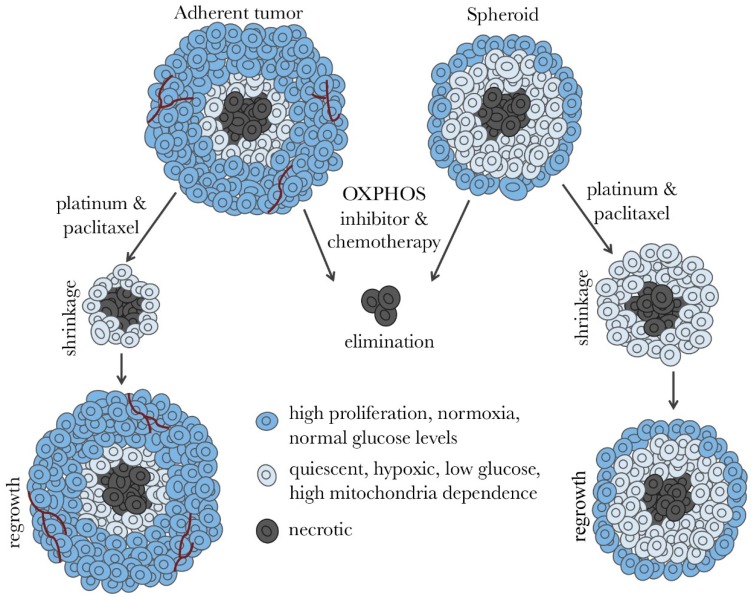
Hypothesis of how standard chemotherapy in combination with OXPHOS inhibitors could be a promising strategy for ovarian cancer elimination and the prevention of reoccurrence. In ovarian tumors and spheroids, standard chemotherapy treatment is very effective at killing highly proliferative cells (dark blue). However, populations of quiescent cells (light blue) survive causing tumor regeneration and cancer reoccurrence. OXPHOS inhibitors are effective at eliminating quiescent ovarian cancer cells in metabolically compromised microenvironments.

Spheroids have a significantly more active TCA cycle than their 2-dimensional counterpart and depend on OXPHOS for ATP production in both quiescent and proliferative layers [46]. A study in breast cancer spheroids showed that OXPHOS was the predominant supplier of ATP in both quiescent and proliferative layers [47]. Increased OXPHOS in ovarian cancer cells enhances IL-6 production [48], which promotes cancer cell survival and proliferation [49], impairs responsiveness to chemotherapy, and shortens progression-free survival of ovarian cancer patients [50]. CSCs have shown high metabolic plasticity prior to differentiation [51,52,53,54] and an increase in TCA cycle activity [46]. Ovarian CSCs have active mitochondria [55,56], higher rates of OXPHOS, and are more sensitive to OXPHOS inhibitors than non-CSC [54,56,57]. Taken together, this supports the notion that spheroid formation, lower proliferation rates, and chemoresistance in ovarian cancer are associated with greater mitochondrial function and dependence on OXPHOS. OXPHOS dependence of quiescent ovarian cancer cells, in combination with the limited potential to switch to glycolysis in glucose-deprived tumor regions, offers the potential for targeted therapies in cooperation with chemotherapy to eliminate proliferative and quiescent cancer cells simultaneously and prevent reoccurrence. See Figure 2 and Figure 3.

## 5. MYC-CDK2/4-RB1 Signaling Pathways

One of the anabolic pathways commonly activated in tumor cells is the MYC pathway [58]. MYC has multiple functions, one of which is to increase oxygen consumption and mitochondrial mass [59]. This regulation may either be direct via binding of MYC to mitochondrial genes and also binds the TFAM gene, a key transcriptional regulator and mitochondrial DNA replication factor [59]. MYC is amplified in Ovarian Clear Cell Carcinoma and alterations in MYC-CDK2/4-RB1 signaling pathways is found in 75% of all tumors in this group [60]. Both disease-free and overall survival of ovarian cancer patients as negatively associated with high MYC expression and high MYC levels are associated with platinum resistance [61]. Interestingly, treatment with MYC-siRNA has been found to result in significant reduction in vivo tumor growth [62]. Whether increased mitochondrial biomass observed in ovarian cancer [41] is correlated to MYC amplification has to our knowledge not been investigated.

## 6. Microenvironment-Independent Mitochondrial Addiction in Ovarian Cancer SWI/SNF Mutations and Metabolism

In addition to microenvironmental factors in ovarian tumors and spheroids leading to quiescence and OXPHOS-dependence, there are also genetic mutations that cause aberrant dependence on OXPHOS in specific subtypes of ovarian cancer [63]. The SWI/SNF nucleosome remodeling complex plays a role in cell proliferation, stemness, and differentiation and is among the most frequent mutations in cancer [64]. Inactivating mutations of ARID1A, a DNA binding subunit of the SWI/SNF complex, are found in over 50% of ovarian clear cell carcinoma (OCCC) cases [65] and 30% of ovarian endometrioid carcinomas [66] and are associated with late stage diagnosis and recurrence. Mutations in SMARCA4, an ATPase and helicase subunit of the SWI/SNF complex, are characteristic features of SCCOHT [67,68], and considered a major cause of the disease. A recent study on lung cancer implicated ARID1A and SMARCA4 in the enrichment of OXPHOS pathways and increased mitochondrial respiration [63]. Furthermore, SMARCA4 and ARID1A mutated tumors were significantly more sensitive to inhibition of OXPHOS as compared with wild-type [63], which opens a therapeutic window for cancer treatment via OXPHOS inhibitors. As previously mentioned, OCCC has a higher rate of recurrence and lower survival rate compared to other types of EOC. Although rare, SCCOHT occurs primarily in young women (median age of 24 years [69]) and is virtually untreatable. Less than 10% of SCCOHT patients survive the first 5 years after diagnosis [7]. OXPHOS inhibition as a targeted treatment for ovarian cancer could be especially impactful on the outcomes of patients with these mutations and subtypes leading to personalized treatments for patients.

## 7. Microenvironment-Independent Mitochondrial Addiction in Ovarian Cancer Mitochondria Motility and Metastasis

In addition to playing a role during cancer cells’ adaptation to hypoxic environment, mitochondria have been shown to be important during solid tumor metastasis and in chemoresistance [70,71,72]. During metastasis, energetically active mitochondria need to reposition from the perinuclear regions where they are normally found, to the cells’ leading edges. This repositioning has been suggested to promote tumor invasion and metastasis via providing the local energy needed for cancer cells to invade the surrounding tissues [70]. Microtubules associated proteins (MAPs) including syntaphilin (SNPH) play a crucial role in mitochondria dynamics by anchoring mitochondria to microtubules thus limiting their trafficking within cells. In this scenario, cells lacking SNPH have more motile mitochondria. While SNPH was originally identified as a regulator or axonal mitochondria, a number of increasing evidences indicate that downregulation of SNPH is a trait of aggressive and metastatic human tumors as it leads to accumulation of mitochondria to cells’ leading edges thus promoting metastasis [73]. According to the Cancer Genome Atlas (https://cancergenome.nih.gov/), in ovarian cancer, the levels of SNPH are 4-fold lower than in the normal ovarian tissues suggesting that abnormal mitochondria positioning may be a contributor of poor patient outcome.

## 8. Drug Screens and Prospective Therapies

Depriving cells of glucose and oxygen in vitro has been shown to reduce the efficacy of several conventional anticancer drugs, including cisplatin [74], which underscores the importance of identifying anticancer agents under conditions of nutrient depravation. Esumi and coworkers have described a number of natural products and other drugs that show anti-proliferative effects on tumor cells grown under conditions of nutrient starvation. Strikingly, all of these drugs interrupt OXPHOS by inhibiting members of the electron transport chain (ETC). In vitro multicellular tumor spheroids (MCTS) have been used in drug screens to identify compounds that are effective against 3-dimensional spheroids. One such screen resulted in the identification of a number of ETC inhibitors (oligomycin A, rotenone, atpenin A5 and metformin) as well as mitochondrial uncouplers that dissipate the proton gradient driving ATPase function (valinomycin, dinitrophenol, and FCCP) [75]. A separate MCTS 16,000-compound screen identified ETC inhibitors pyrvinium pamoate and salinomycin, and uncouplers closantel, nitazoxanide, and niclosamide [76]. A 10,000-compound MCTS screen identified one novel OXPHOS inhibitor, VLX600, which reduces the viability of quiescent cells in in vivo tumors as well as causing spheroid reduction, and inhibition of tumor proliferation [77]. In summary, drug screens under nutrient starvation conditions and screens using MCTS have consistently identified candidates that interrupt OXPHOS by inhibiting or uncoupling the ETC.

Many of the mitochondrial inhibitors from the aforementioned drug screens are antibiotic or anti-parasitic agents. Antibiotics such as macrolides, clindamycin, tetracycline, chloramphenicol, and linezolid have an inhibitory effect on the expression of ETC complexes. Azithromycin has been shown to enhance the effects of cisplatin and paclitaxel [78] and inhibit sphere formation [79]. Doxycycline inhibits the proliferation of ovarian cancer cells and is selectively potent against cisplatin-resistant cells [80]. Salinomycin inhibits growth, decreases viability, and promotes apoptosis of ovarian CSCs in combination with paclitaxel [81,82]. Alone, salinomycin has an apoptotic affect that selects for ovarian cancer cells, but not normal ovarian cells [83] and is more potent against cisplatin-resistant ovarian cancer cell lines than cisplatin-sensitive. Bedaquiline inhibits ATP-synthase in bacteria and mitochondria [84] and has been shown to hinder CSCs [85]. While not a direct inhibitor of mitochondrial activity, the activator of pyruvate dehydrogenase (PDH), CPI-613 has been shown to indirectly suppress mitochondrial ATP production and lead to cell death in several cancer cell lines including ovarian cancer cells [86].

Clinically available drugs, including antibiotics, are attractive options for exploring alternative treatments for ovarian cancer because their toxicity and dosing are already understood; so too is the diabetes medication, metformin, which inhibits Complex I of the ETC [87]. Systemic review of metformin use and ovarian cancer showed a 40% reduction in cancer incidence and a 60% improvement in survival, however, the studies reviewed were primarily observational and the authors suggest these results should be interpreted cautiously [88]. In-vivo, metformin alone inhibits cell proliferation [89,90] tumor growth, metastasis and angiogenesis [89]. Combination with cisplatin enhanced the effect [91] and combination with paclitaxel showed a 60% reduction in tumor mass [90]. In Table 1 are examples of mitochondrial inhibitors reported to have antineoplastic activity in different tumor models. At least three of these inhibitors have been entered into clinical Phase 1 trials (Clinicaltrials.gov). Results on inhibition of tumor progression are generally not available or have been included as endpoints of the studies. The complex I inhibitor BAY 87-2243 has been evaluated in a Phase 1 study (NCT01297530) but results were not reported. Niclosamide was concluded not to be a viable compound for treatment of colorectal cancer due to toxicity (NCT02532114) [92]. Finally, a number of Phase 1 studies are ongoing or have been conducted with metformin with results generally not reported. One of the studies where have results were reported is NCT01579812 (“Evaluation of Metformin, Targeting Cancer Stem Cells for Prevention of Relapse in Gynecologic Patients”). Only 38 patients were evaluated in the study where metformin was combined with chemotherapy, too few in order to address the question of whether metformin will indeed improve the clinical course of ovarian cancer.

## 9. Conclusions

Late stage ovarian cancer remains an aggressively recurrent disease. Ovarian tumors grow to be very large and shed malignant cells into the ascites that organize into compact cellular spheroids capable of adhering to peritoneal tissues and forming metastatic tumors. Depletion of environmental oxygen and glucose arises due to irregular vascularization of tumors and confinement to the peritoneal cavity. The standard chemotherapy treatment of carboplatin and paclitaxel is effective at eradicating proliferative cells, but leaves behind populations that can cause the disease to reoccur. Recurring patients have widely metastatic disease, decreased response to future chemotherapy and almost always ultimately succumb to it. Effective treatment of late stage ovarian cancer remains a major challenge.

In order to tackle the problem of recurrence in ovarian cancer, chemoresistant cell populations must be characterized and their weaknesses exploited. Since chemotherapy targets rapidly proliferating cells, quiescent cells are immune to its effects. The microenvironment of ovarian cancer is hypoxic and glucose deprived due to irregular vascularization. Quiescent tumor cells depend on OXPHOS for the majority of energy production. Due to their state of nutritional starvation the capacity for metabolic plasticity is limited, which sensitizes them to mild inhibition of their major source of ATP: OXPHOS. OXPHOS inhibition could also improve our ability to target CSCs, which are major contributors to recurrence. Highly chemoresistant and present throughout ovarian tumors, ascites, and spheroids, CSCs depend on metabolic plasticity to survive adverse microenvironments. Accordingly, they have hyperactive mitochondria and are highly dependent on OXPHOS. Furthermore, subtypes of ovarian cancer that are particularly difficult to treat have a genetic enhancement of OXPHOS activity. The aberrant dependence on OXPHOS in ovarian cancer calls for therapeutic improvements with inhibitors of mitochondrial respiration that allow a therapeutic window for normal cells. Such a threshold has been demonstrated in cells for several OXPHOS inhibitors. Recently, mitochondria spatial redistribution has also been associated to tumor metastasis.

In this scenario, antibiotics that disrupt the ETC such as azithromycin, doxycycline, salinomycin, and bedaquiline have demonstrated anti-cancer activity including inhibition of spheroid formation, growth inhibition of chemoresistant cells, and elimination of CSCs [78,79,80,81,82,83,84,85]. The commonly-used diabetes drug and ETC inhibitor, metformin, has had a remarkable anti-cancer effects in ovarian cancer cells and CSCs and is suggested to dramatically improve survival rates in diabetic ovarian cancer patients [88,91]. As previously mentioned, many of these drugs significantly improve the effects of standard ovarian cancer treatment. Use in combination with carboplatin and/or paclitaxel could lead to major improvements in outcomes of ovarian cancer patients.

## Figures and Tables

**Table 1:** Examples of mitochondrial inhibitors reported to have antineoplastic activity.

	Target	Effect in Tumor Model	References
Salinomycin	Mitochondrial K+/H+ Exchange [93]	Colon cancer	[94]
		Pancreas cancer, combination	[95]
		Ovarian cancer	[96]
		Nasopharyngeal carcinoma	[97]
		Colon cancer	[98]
Nitazoxanide	Uncoupler [76]	Colon cancer, combination	[76]
		Breast cancer	[99]
Niclosamide	Uncoupler [100]	Colon cancer metastasis model	[100]
		Radiotherapy-resistant breast cancer	[101]
		Adrenocortical carcinoma	[102]
		Breast cancer	[103]
Bedaquiline	OXPHOS [85]	Lung cancer	[84]
VLX600	OXPHOS [77]	Colon cancer	[77]
		Gastrointestinal stromal tumor	[104]
Pyrvinium	OXPHOS [79]	Pancreas cancer	[105]
Metformin	OXPHOS [106]	Ovarian cancer	[89]
		Ovarian cancer	[90]
		Ovarian cancer	[91]
		Glioblastoma	[107]
		Colon cancer	[108]
BAY 87-2243	Complex I [109]	Melanoma	[110]
Atovaquone	Complex III [111]	Head-neck cancer, radiation enhancement	[111]
Azithromycin	Mitochondrial protein synthesis [79]	Lung cancer	[112]
Doxycycline	Mitochondrial protein synthesis [79]	Ovarian cancer, peritoneal	[80]
		Breast cancer bone metastasis	[113]
Tigecycline	Mitochondrial protein synthesis [114]	Lung cancer	[115]
		Hepatocellular carcinoma	[116]
	Mitochondrial protein synthesis [114]	Lung cancer	[115]
		Hepatocellular carcinoma	[116]
